# Construction and development of an auto-regulatory gene expression system in *Bacillus subtilis*

**DOI:** 10.1186/s12934-015-0341-2

**Published:** 2015-09-21

**Authors:** Chengran Guan, Wenjing Cui, Jintao Cheng, Li Zhou, Junling Guo, Xu Hu, Guoping Xiao, Zhemin Zhou

**Affiliations:** School of Biotechnology, Key Laboratory of Industrial Biotechnology (Ministry of Education), Jiangnan University, 1800 Lihu Road, Wuxi, Jiangsu 214122 China; Wuxi Biortus Bioscience Co., Ltd, Wuxi, Jiangsu 214122 China

**Keywords:** *Bacillus subtilis*, Protein expression system, Promoter, Quorum sensing, Auto-inducible, Auto-regulatory, Over-production, *srf* operon

## Abstract

**Background:**

*Bacillus subtilis* is an all-important Gram-positive bacterium of valuable biotechnological utility that has been widely used to over-produce industrially and pharmaceutically relevant proteins. There are a variety of expression systems in terms of types of transcriptional patterns, among which the auto-inducible and growth-phase-dependent promoters are gaining increasing favor due to their inducer-independent feature, allowing for the potential to industrially scale-up. To expand the applicability of the auto-inducible expression system, a novel auto-regulatory expression system coupled with cell density was constructed and developed in *B. subtilis* using the quorum-sensing related promoter *srfA* (P_srfA_).

**Results:**

The promoter of the *srf* operon was used to construct an expression plasmid with the green fluorescent protein (GFP) downstream of P_srfA_. The expression displayed a cell-density-dependent pattern in that GFP had a fairly low expression level at the early exponential stage and was highly expressed at the late exponential as well as the stationary stages. Moreover, the recombinant system had a similar expression pattern in wild-type *B. subtilis* 168, WB600, and WB800, as well as in *B. subtilis* 168 derivative strain 1681, with the complete deletion of P_srfA_, indicating the excellent compatibility of this system. Noticeably, the expression strength of P_srfA_ was enhanced by optimizing the −10 and −35 core sequence by substituting both sequences with consensus sequences. Importantly, the expression pattern was successfully developed in an auto-regulatory cell-density coupling system by the simple addition of glucose in which GFP could not be strongly expressed until glucose was depleted, resulting in a greater amount of the GFP product and increased cell density. The expression system was eventually tested by the successful over-production of aminopeptidase to a desired level.

**Conclusion:**

The auto-regulatory cell density coupling system that is mediated by P_srfA_ is a novel expression system that has an expression pattern that is split between cell-growth and over-expression, leading to an increase in cell density and elevating the overall expression levels of heterologously expressed proteins. The broad applicability of this system and inducer-free expression property in *B. subtilis* facilitate the industrial scale-up and medical applications for the over-production of a variety of desired proteins.

## Background

*Bacillus subtilis*, a rod-shaped Gram-positive soil bacterium, has been regarded to be a “generally recognized as safe” (GRAS) microbe that can naturally secrete numerous extracellular proteins. These properties make this bacterium to be one of the most popular hosts for naturally producing a variety of enzymes and over-expressing a large number of pharmaceutical and industrial recombinant proteins of interest, such as amylase [1–4], lipase [5], alkaline PGL [6], and so on. The major advantages of *B. subtilis* compared to other protein expression hosts are that the host has high-cell-density growth and can secrete proteins directly into the cultural medium, which greatly simplifies the following steps for purification and preparation.

The most commonly used expression systems in *B. subtilis* are inducible expression systems that contain inducer-specific promoters, such as the T7 promoter; *grac* promoter; *spac* promoter induced by isopropyl-β-d-thiogalactopyranoside (IPTG); *xyl*A promoter driven by xylose; *SacB* promoter induced by sucrose; and α-amylase promoter transcribed in response to starch [4, 7, 8]. Although a variety of homologous and heterologous proteins have been successfully over-produced in *B. subtilis* under the control of these inducible promoters, a noticeable basal expression level was observed from these expression systems. Moreover, the high cost of inducers that need to be added to the medium during fermentation at a large scale would limit the industrial utilization of these protein expression systems. Recently, two strictly inducible systems, the subtilin-regulated expression (SURE) system and the LiaRS-controlled expression system (LIKE), which rely on the regulation of the promoter for the spa-box and the cell envelope stress-responsive *liaI* promoter, respectively, have been developed for scientific and industrial applications. Both of these protein expression systems are strictly controlled by the addition of lantibiotic subtilin and bacitracin, respectively, after the exponential phase of cell growth, preventing the leakage of transcription under non-inducing conditions [9, 10].

To avoid the addition of any inducers, promoters under the control of a variety of environmental stresses, such as heat shock, salt, acid, and ethanol stresses, as well as oxygen or glucose starvation [11–13], have also been used to construct systems for the expression of a variety of proteins in *B. subtilis*, none of which, could be used to over-produce industrially and pharmaceutically utilized proteins and enzymes because of the low and non-persistent expression levels of these systems as well as the difficulty of controlling protein expression. Very recently, an artificial auto-inducible system using a growth-phase promoter (*cry3Aa* promoter, transcribed during the stationary phase) and another expression system using the nutrient starvation response promoter (*pst* promoter, responded to phosphate starvation) [14, 15] were developed and characterized. Importantly, a self-inducible system for the reliable and low-cost over-production of recombinant proteins has been developed by using the manP-encoding phosphotransferase system (PTS), which is under the control of the relatively strong and strict promoter manP; manP is transcribed during a glucose-limited process in the engineered *B. subtilis* strain TQ356 [16]. The self-controlled system is suitable for high-cell-density fermentation because cell growth and over-expression are discrete at different stages.

**Fig. 1:**
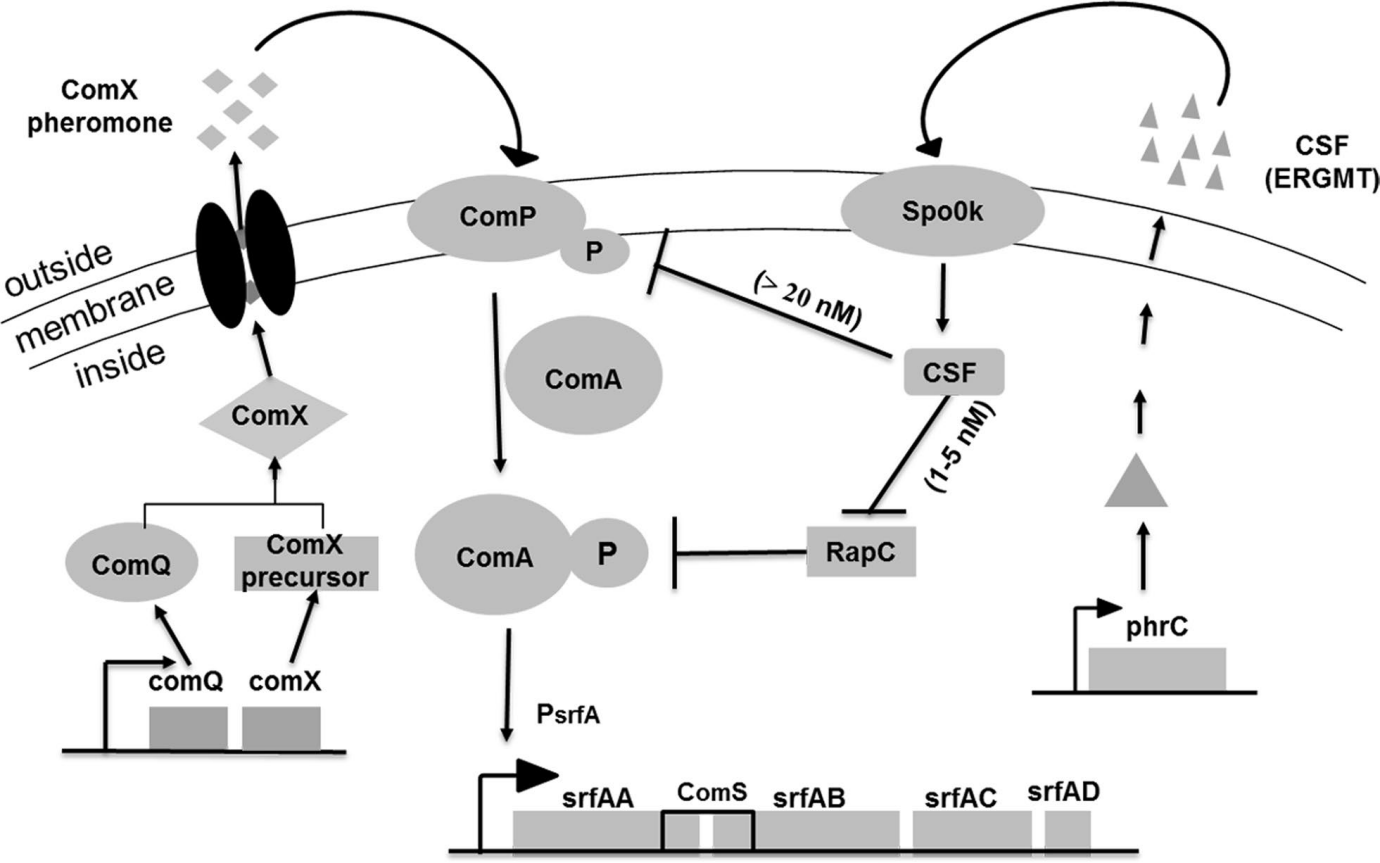
The schematic model for the regulation of the transcription of the *srf* operon network involved in two extracellular signaling peptide-mediated quorum sensing in *B. subtilis*. The *srf* operon was composed of four genes encoding surfactin synthetase with *comS* embedded between gene *srfAA* and *srfAB*. Both ComX and CSF activate the transcription of the *srf* operon by stimulating the activity of transcription factor ComA through phosphorylation (ComA-P) via two separate pathways, one of which requires the ComX peptide to be processed outside by ComQ. Another pathway is mediated by CSF, which is encoded by *phrC* and is initially imported as inactive-form CSF (inact). The active-form CSF (act), which interacts with two intracellular targets, is generated through processing by the *Spo0K* gene. At low concentrations, CSF inhibits the activity of an aspartyl-phosphate phosphatase, RapC, and is speculated to inhibit the kinase activity of ComP at high concentrations

Bacterial quorum sensing (QS) systems are cell-density-dependent regulatory networks that coordinate bacterial behavioral changes from single cellular organisms at low cell densities to multicellular organisms when their population density reaches a threshold level. The two peptides, ComX and CSF, mediate the quorum sensing control of competence and sporulation in *B. subtilis*. The accumulation of a high concentration of peptides in the late-growth phase triggers a series of gene transcription events downstream of the ComA-ComP two-component system in the cell [17], among which the *srf* operon is stimulated via a complex-regulation phosphorylated cascade (Fig. 1) [18]. As a monitor of cell density, the ComX pheromone is expressed, transported and accumulated in the broth along with cell growth. When the cells reach a certain density, the ComX pheromone reaches the obligatory concentration to activate the signal transduction system, which is composed of the two-component regulatory proteins ComP and ComA, and finally, phosphorylated ComA binds to the promoter (P_srfA_) of the *srf* operon to activate the transcription of corresponding gene expression [19–21], leading to temporally controllable transcription.

Inspired by the transcriptional pattern of P_srfA_, a novel gene expression system in *B. subtilis* was developed and characterized by harnessing the transcriptional properties of P_srfA_ as controlled through the response to cell density and glucose concentration during culturing. Gene expression under the control of the promoter was significantly inhibited via the addition of an appropriate concentration of glucose at the cell-growth phase until glucose was depleted, permitting the coupling of gene expression to cell density and the concentration of glucose. The functionality was examined by the efficient and controllable expression of GFP and aminopeptidase (AP). This auto-regulatory expression system could be used as an efficient tool for gene over-expression in *B. subtilis*.

## Results and discussion

### Expression pattern of GFP under the control of P_srfA_ in *B. subtilis* 168

**Fig. 2:**
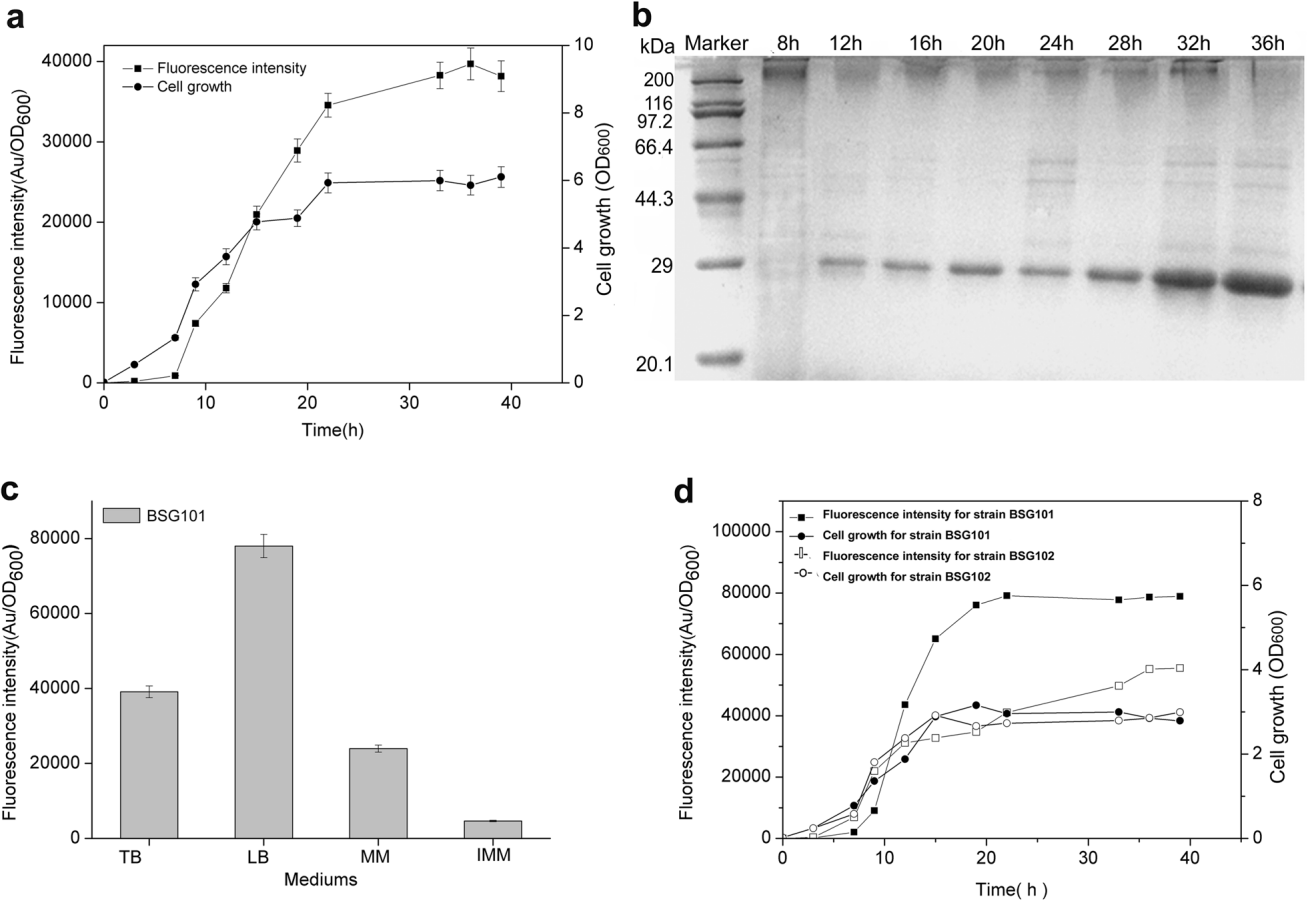
Identification of the expression level and patterns of GFP in recombinant *B. subtilis* 168. **a** The fluorescence intensity of GFP (*solid rectangle*) and cell growth (*solid cycle*) under the control of wild-type P_srfA_ were monitored in recombinant *B. subtilis* 168 during cultivation in TB medium. **b** SDS-PAGE analysis for the expression of GFP in *B. subtilis* 168 harboring pBSG03 with P_srfA_, which was cultivated in TB medium, showed the expression pattern. Samples were collected periodically at different time points, and total proteins of 30 μg were loaded onto the gel. **c** The impact of the medium on the expression level of GFP in *B. subtilis* 168 harboring pBSG03 (BSG101). The expression level of GFP under the control of P_srfA_ was determined by the total fluorescence intensity at OD_600_ (denoted as Au/OD_600_). The recombinant strain was cultivated in TB, MM, IMM and LB medium. All of the data were collected in triplicate and were presented as the mean ± SD. **d** Comparison of the expression patterns and levels controlled by P_sfrA_ and P_HpaII_. *B. subtilis* 168 harboring pBSG03 (BSG101) and pBSG04 containing the P_HpaII_ promoter (BSG102) were cultivated in LB medium for 40 h. The fluorescence intensity and cell growth for each of the recombinant strains were examined by periodic sampling throughout cultivation. The cell density of BSG101 (*solid cycle*) and BSG102 (*open cycle*) was monitored by measuring the OD_600_. The fluorescence intensity of BSG101 (*solid rectangle*) and BSG102 (*open rectangle*) was monitored throughout the process at corresponding time points

In recent years, several types of expression systems that are free of inducers via a variety of transcriptional patterns have been developed in *B. subtilis* or other Gram-positive hosts to over-express industrially and pharmaceutically used proteins [13, 15, 16]. To expand the application of self-inducible or auto-inducible gene expression systems in *B. subtilis*, a novel recombinant plasmid, pBSG03, harboring a cell-density-responsive promoter, P_srfA_, upstream of the reporter protein GFP was constructed, permitting the monitoring of gene expression in the hosts. To characterize the expression pattern and strength for P_srfA_, pBSG03 was transformed into *B. subtilis* 168 and then cultivated in Terrific Broth (TB) medium for 40 h, during which the culture was sampled periodically to examine the expression level of GFP. The results demonstrate that the expression level of GFP was considerably low from the lag phase to the early exponential phase. Subsequently, the expression level increased sharply when cell growth entered the late exponential phase. Thereafter, the expression level increased gradually throughout the stationary phase until the relative fluorescence unit reached the vertex (Fig. 2a). In parallel, the intracellular proteins that were sampled in a time course were determined by SDS-PAGE analysis. The data indicated that the expression of GFP quantitatively was accumulated 9 h after fermentation, before which there were few GFP proteins that were scarcely detected on the PAGE (Fig. 2b). These results indicate that the transcription of P_srfA_ occurs in the late growth phase, whereas the activity of the promoter is inhibited in the early stage of cell-growth, permitting cell-density-dependent expression. The cell-density-dependent transcription of P_srfA_ is subjected to simple regulated mechanism of the ComA-ComP phosphorylation cascade compared to that of the recently reported gene expression system, by which the P_manP_ is involved in a rather complex regulatory network, making it necessary to delete the *manP* gene in the operon to achieve self-inducible expression properties. Otherwise, the gene expression under P_manP_ is typically mannose-inducible in wild-type *B. subtilis* [16].

Meanwhile, the effect of four different culture media (TB medium, MM medium, IMM medium and LB medium) on P_srfA_-mediated GFP expression in *B. subtilis* 168 (BSG101) was estimated in the following experiments. The data of the fluorescence intensity showed that the recombinant strain grown in LB medium achieved the maximal expression level, in which the expression level of GFP was approximately twofold higher than that in TB medium (Fig. 2c).

P_HpaII_ is a strong constitutive promoter, has comparable expression strength to that of P_43_, and has been used to construct an array of vectors for the over-production of recombinant proteins [22, 23]. To contrast the transcriptional level of P_srfA_ with that of P_HpaII_, plasmid pBSG04 harboring the P_HpaII_-GFP expression cassette was constructed and transformed into *B. subtilis* 168, generating BSG102. Over the 40 h of fermentation in LB medium, each culture of the two recombinant strains was sampled periodically to measure the expression level. The data showed that the overall expression of GFP in BSG101 was higher than that of BSG102 by 1.5-fold (Fig. 2d), even though the final biomasses of the two strains were nearly the same. These results indicate that the P_srfA_ is a potentially strong promoter for gene expression in *B. subtilis*.

### Expression pattern of GFP in markerless deletion mutant and the protease-deficient mutants

Based on the organization of the *srf* operon, the native P_srfA_ would also be activated, resulting in the expression of genes in the operon. To ascertain whether the background activation of the *srf* operon occurred simultaneously with heterologous gene expression, the markerless deletion mutant BSG1681 was constructed by completely deleting the full-length of P_srfA_ on the chromosome.

**Fig. 3:**
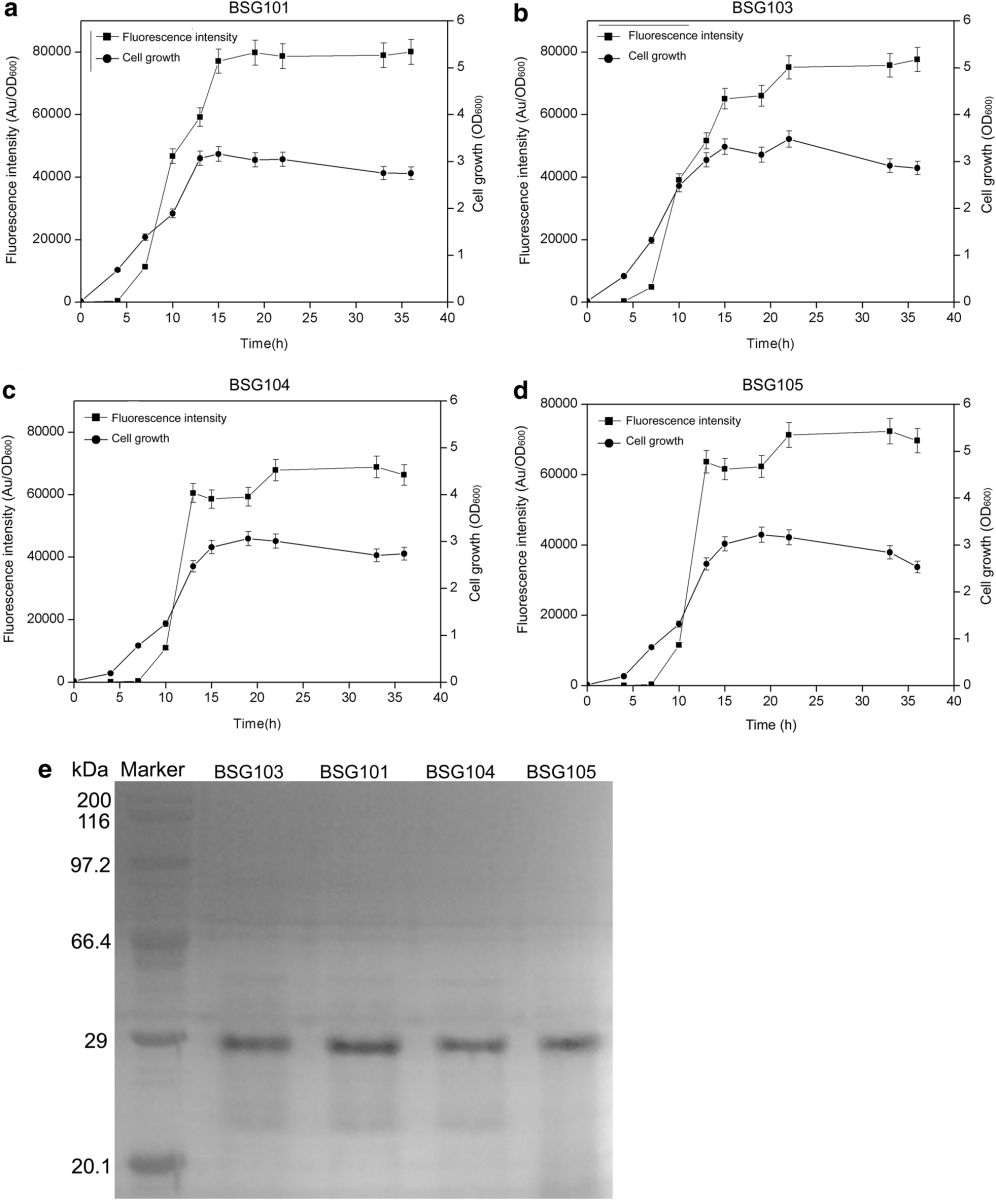
Over-expression of GFP using the plasmid pBSG03 in *B. subtilis* 168 (**a**), BSG1681 (**b**), *B. subtilis* WB600 (**c**), and *B. subtilis* WB800 (**d**) was compared by determining the cell density and relative fluorescence intensity (Au/OD_600_). The final expression level of GFP in the different four recombinant *B. subtlis* strains was demonstrated by SDS-PAGE analysis (**e**). The expression of GFP in BSG101, BSG103, and BSG105 was approximately equal, but slightly higher than that in BSG104

To compare the transcriptional features and expression levels of P_srfA_ in BSG1681 with the other three types of hosts (wild-type 168, *B. subtilis* WB600 and WB800), plasmid pBSG03 was introduced into the above hosts, generating BSG103, BSG101, BSG104, and BSG105, respectively. The levels of fluorescence intensity were monitored in the four recombinant strains throughout culturing. As expected, the transcriptional feature of P_srfA_ in BSG103 was similar to that in BSG101, BSG104 and BSG105, giving a transcription pattern of cell-density-dependent auto-regulation. Noticeably, the final fluorescence intensity in BSG101, BSG103 and BSG105 was similar, but slightly higher than that in BSG104 (Fig. 3a–d). These results suggest that P_srfA_ has a broad transcriptional compatibility with the cell-density-dependent pattern in various hosts. The final expression levels of GFP in the four different hosts were analyzed by SDS-PAGE, and the data were consistent with those of the fluorescence intensity (Fig. 3e).

### Enhancement of the expression level by engineering the consensus sequence within the *srfA* promoter

Although the cell-density-dependent expression system was characterized as highly transcribed and broadly compatible in various *B. subtilis* hosts, the duration of exponential expression under the control of the wild-type promoter was short, scarcely 10 h (Fig. 3a–d). The exponential expression phase in the other constructed auto-regulation systems starts at approximately the mid-exponential growth phase and terminated during the beginning of the stationary phase [24]. In addition, the activity of the wild-type promoter (e.g., *cry3Aa*), which behaves in a cell-density-dependent manner, usually disallows the over-production of a large amount of heterologous proteins to the desired level. Thus, engineering the promoters for higher capacity is the major demand for augmenting the over-production of heterologous proteins [9].

**Fig. 4:**
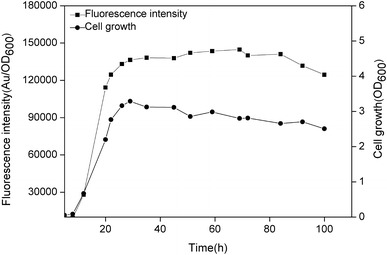
The expression level of GFP controlled by the mutant P_srfA_ (^mut^P_srfA_) in *B. subtilis* 168. The recombinant strain harboring pBSG05 was constructed to analyze the alteration of the transcription and expression pattern. During 100 h of cultivation, cells were sampled periodically and analyzed by examining the cell density and fluorescence intensity

As one of the most efficient and prevalent strategies, promoter engineering has been widely used to tune the gene expression level, both for high levels of protein expression and tunable gene expression in synthetic biology [25, 26]. The major essential elements in prokaryotic promoters that mainly determine their overall transcriptional activity are the −10 and −35 consensuses motifs, while other factors, such as the gene context, could also influence transcriptional activity [27]. This feature indicates that the two motifs are potential targets to be engineered within the promoter sequence. In this study, to enhance the activity of P_srfA_, the sequence of the −10 and −35 motifs in P_srfA_ was compared with the σ^A^-dependent consensuses in *B. subtilis*. The alignment showed that the two motifs in P_srfA_ were in poor homology to the consensus. Therefore, the consensus −10 (TATAAT) and −35 (TTGACT) motifs of σ^A^-dependent promoters substituted for the −10 (TAAACT) and −35 (GTGATA) motifs of the P_srfA_ in plasmid pBSG03, resulting in plasmid pBSG05. After transforming pBSG05 into *B. subtilis* 168 (generating BSG106), the cell growth and fluorescence intensity were monitored throughout culturing. After 20 h of culture, the fluorescence intensity peaked, and thereafter, the maximal level did not decrease until 80 h of culture. The maximal expression level under the control of engineered P_srfA_ was approximately twofold stronger than that of the wild-type promoter (Fig. 4), demonstrating that engineering the poorly conserved −10 and −35 motifs within the promoters lead to fold increases of the transcriptional level. Despite engineering the non-inducible promoters, a set of other promoters, including inducible promoters, has been engineered to achieve a stronger over-production level through this strategy [15, 28].

### Development of the cell-density-dependent expression system to an auto-regulatory system

The aforementioned expression system displayed a cell-density-dependent expression pattern. However, the cell density did not achieve a higher level than expected. According to the data referring to the expression pattern, we proposed that the initiation of a high expression level overlapped the rapid growth stage, leading to competition for energy and chemicals. Therefore, to achieve a high cell density and the desired over-production, expression initiation should be retarded until the cell density reaches a high level, avoiding mutual intervention between the two biological events. Nutrients that delayed expression while promoting cell growth were regarded as prospective candidates. A previous study revealed that the addition of glucose to the growth medium inhibits the expression of many genes, including *srf* operon [29].

**Fig. 5:**
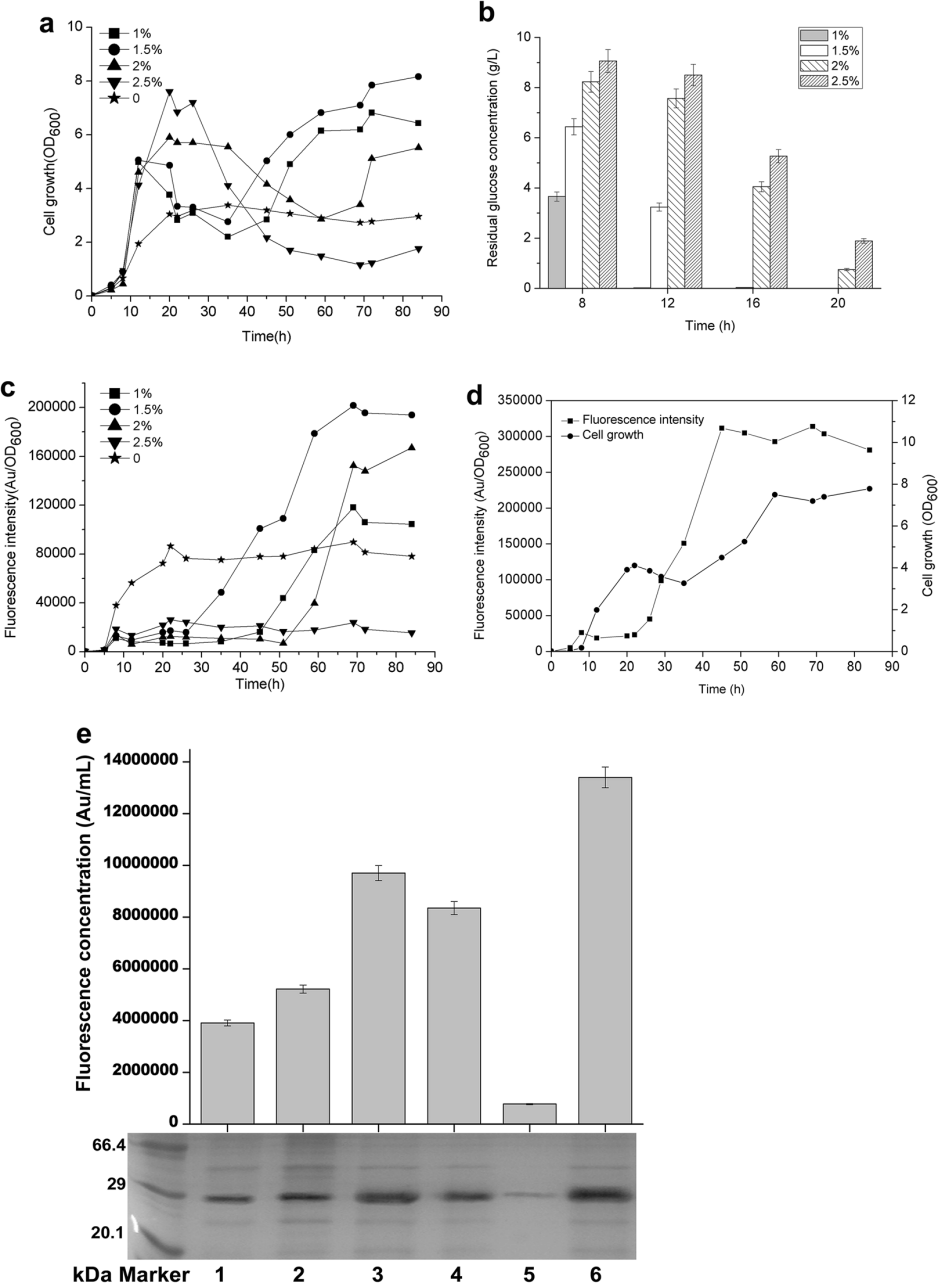
The effect of different concentrations of glucose on the P_srfA_- and ^mut^P_srfA_-controlled expression of GFP. **a** The cell density profiles for BSG101 harboring wild-type P_srfA_ in LB medium with the addition of 1 % (*solid rectangle*), 1.5 % (*solid cycle*), 2 % (*solid triangle*), and 2.5 % (*solid inverse triangle*) glucose. Medium without the addition of glucose was used as the control group (*solid pentangle*). **b** Glucose consumption profiles at 8, 12, 16, and 20 h with different initial concentrations. **c** The fluorescence intensity profiles that were detected in BSG101 with different concentrations of glucose corresponded to those in **a**. **d** The fluorescence intensity and the cell density that were detected in recombinant strain BSG106 that harbored ^mut^P_srfA_ in which the intrinsic sequences of −10 and −35 were replaced by the corresponding conserved sequences for σ^A^-dependent promoters. **e** Comparison of the highest fluorescence intensity and the corresponding GFP expression level with or without the addition of glucose in *B. subtilis* 168 under the control of wild-type P_srfA_ and ^mut^P_srfA_. Sample 1 represents the BSG101 without the addition of glucose, which was designated as the control. Samples 2–5 represent strain BSG101 with the addition of 1, 1.5, 2, and 2.5 % glucose, respectively. Sample 6 represents strain BSG106 (harboring ^mut^P_srfA_) with the addition of 1.5 % glucose. All of the experiments were independently performed in triplicate

Thus, various concentrations of glucose were chosen to appraise the influence of glucose on the expression pattern, cell biomass and expression levels. First, the cell growth, fluorescence intensity and glucose consumption rate were measured with the addition of 1, 1.5, 2, and 2.5 % concentrations of glucose. The addition of all of the concentration of glucose, except for 2.5 %, elevated the final cell-density in the BSG101 strain; the addition of 1.5 % glucose led to the highest cell density, which was nearly threefold higher than that of the glucose-free culture (Fig. 5a). The 1.5 % glucose in the medium was depleted after 16 h, at which time the 2 and 2.5 % glucose remained in the medium (Fig. 5b). GFP expression sharply increased in the 1, 1.5, and 2 % glucose groups when glucose was depleted, before which GFP was significantly inhibited (Fig. 5c). Among the four groups, the highest expression level of GFP was acquired by the addition of 1.5 % glucose. The highest expression level was more than 2.5-fold higher than that of glucose-free cultivation (Fig. 5c), indicating that the addition of glucose not only augment the expression level of heterologous genes but also give the system an auto-regulatory expression pattern. Interestingly, the addition of 2.5 % glucose completely inhibited expression throughout cultivation (Fig. 5c). Then, the expression of GFP in BSG106 was examined to determine the effect of 1.5 % glucose on the expression level of GFP under the engineered P_srfA_ (^mut^P_srfA_). As expected, the expression of GFP initiated after the 1.5 % glucose was depleted and maintained at the maximal level from 40 h until the end of cultivation (Fig. 5d). The maximal expression level under the control of ^mut^P_srfA_ was approximately fourfold higher than that of the wild-type promoter P_srfA_ without adding of 1.5 % glucose (Fig. 2d)_._ Moreover, the total amount of GFP was determined by measuring the average fluorescence intensity (as calculated by arbitrary units per milliliter of culture) and by SDS-PAGE analysis. The highest fluorescence intensity and accumulative GFP protein were obtained under the control of ^mut^P_srfA_ with the addition of 1.5 % glucose (Fig. 5e).

Taken together, the cell-density-dependent system that is mediated by P_srfA_ could be contrived to be an auto-regulatory device by the regulatory effect of glucose repression. This simple method eventually achieves a high cell density without a complex gene deletion or modification of the genome in the host. Regarding other developed auto-inducible expression systems, certain genes that are involved in the regulatory network should be disrupted or deleted to make an auto-inducible expression system while preventing native gene expression from interfering with the heterologous transcription that is mediated by the used promoters. Generally, *B. subtilis* under the control of P_manP_, which is intrinsically a mannose-induced system, has been successfully fabricated to an auto-inducible expression by the deletion of the *manP* gene in the PTS pathway [16].

### Over-production of AP using the auto-regulatory gene expression system

**Fig. 6:**
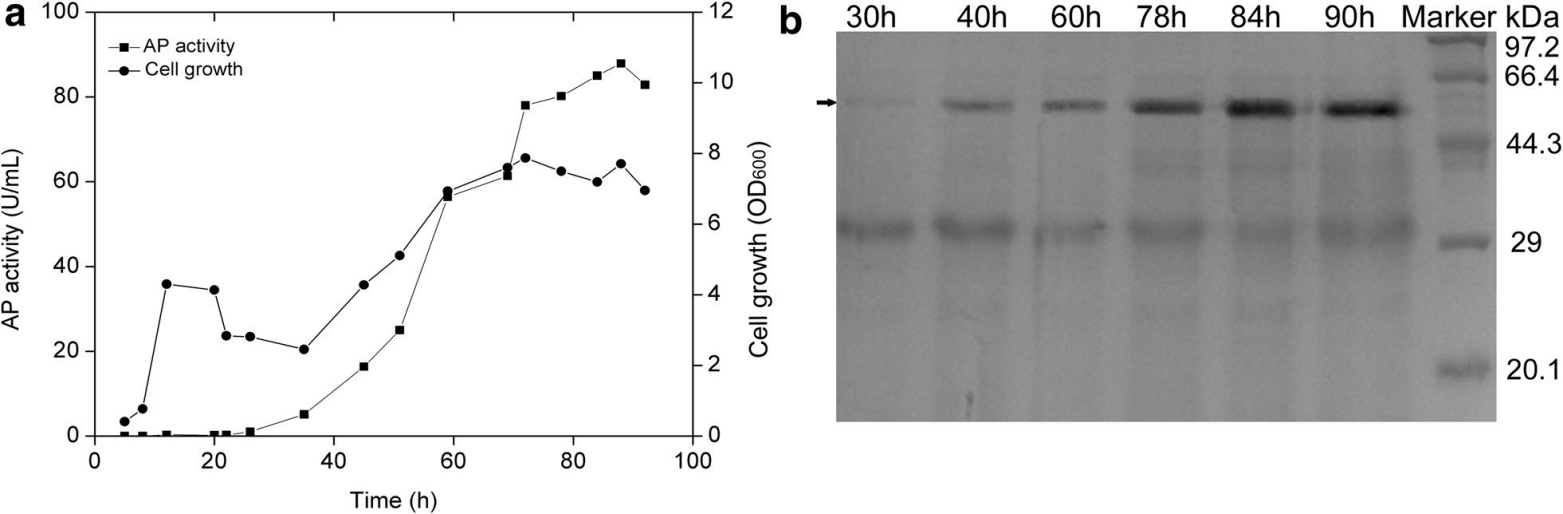
Over-production of aminopeptidase (AP) using the constructed auto-regulatory gene expression system. Plasmid pBSG06 harboring the signal peptide (SP_ap_) and AP downstream of the ^mut^P_srfA_ was introduced into *B. subtilis* 168. The recombinant strain BSG107 was then cultured in LB medium for more than 40 h with periodical sampling. **a** The activities of AP in the supernatant (*solid rectangle*) and cell density (*solid cycle*), as determined at different times, displayed an AP over-production pattern that was related to cell growth. **b** The expression of AP was analyzed by SDS-PAGE. The position of the AP protein bands corresponding to 30, 40, 60, 78, 84, and 90 h is marked with a *black arrow*

To estimate the suitability of the system for the over-production of the heterologous protein, AP from *B. subtilis* Zj016, plasmid pBSG06 was constructed under the control of promoter ^mut^P_srfA_. A previous report demonstrated the expression level of AP in *B. subtilis* WB600 under the control of P_HpaII_. However, the expression and secretion levels were incomparable to those of other proteins [22, 30, 31]. Plasmid pBSG06 was introduced into *B. subtilis* 168, generating recombinant strain BSG107. The culture procedure was similar to that for the auto-regulatory expression mentioned above. The cell growth, AP activity, and protein over-production were measured throughout cultivation. As expected, AP expression displayed the auto-regulatory pattern coupled with a cell-density-dependent manner. The highest activity of AP that was obtained was 87.89 U/mL, and the cell-density at OD_600_ achieved a level of approximately 7.87 (Fig. 6a). The expression property also be verified at the protein level by SDS-PAGE analysis (Fig. 6b). Those results indicate that ^mut^P_srfA_-mediated system could be used to express heterologous proteins in *B. subtilis*.

## Conclusions

In this study, a novel auto-regulatory gene expression system coupled to cell density in *B. subtilis* was constructed and developed using the *srfA* promoter that is associated with triggering quorum sensing. Using the expression system, expression was separated by the cell growth phase and high expression phase, during which the addition of glucose not only augmented the two-phase expression profile and enabled auto-regulation but also increased the final cell density. In addition, the expression strength was remarkably enhanced by engineering the −10 and −35 region via substitution to the highly conserved sequences. Furthermore, aminopeptidase was successfully over-produced as the test protein using the newly constructed gene expression system, achieving a 1.7-fold over-expression level compared to that under the control of the generally regarded strong promoter P_HpaII_ [30]_._ These features of the auto-regulatory gene expression system give the system great potential for the over-production of enormous high-value-added proteins at the industrial scale.

## Methods

### Strains, plasmids and growth conditions

The bacterial strains and plasmids that were used in this study are listed in Table 1. *E. coli* JM109 was used as a host for gene cloning. *B. subtilis* 168 and engineered *B. subtilis* BSG1681 were used for the gene expression and gene integration experiments. The media used in this study were glucose minimal medium (MM) [32], improved minimal medium (IMM, adding 0.02 % Casamino acids to MM medium), Luria–Bertani medium (LB), and TB. All of the strains were cultured in the appropriate medium with aeration at 37 °C. When appropriate, *B. subtilis* growth media was supplemented with kanamycin (5 μg/mL) and *E. coli* growth media with ampicillin (10 μg/mL). The cell density was determined by measuring the OD_600_ with a UV-1800/PC spectrophotometer (Shanghai, MAPADA Instrument Co., Ltd., China).

**Table 1:** Strains and plasmids used in this study

Strains and plasmids	Relevant properties	References
Strains		
*E. coli* JM109	*recA*1, *endA*1, *gyrA*96, *thi*-1, *hsdR*17(r_k_^-^m_k_^−^), *e*14^−^(*mcrA* ^−^), *supE*44, *relA*1, Δ(*lac*-*proAB*)/F′ [*traD*36, *proAB* ^+^, *lacI* ^q^, lacZΔM15]	
*B. subtilis*		
168	*trpC2*	
WB600	(168) Δ*nprE*, Δ *aprA*, Δ*epr*, Δ *bpr*, Δ*mpr*, Δ*nprB*	[40]
WB800	(168) Δ*nprE*, Δ *aprA*, Δ*epr*, Δ *bpr*, Δ*mpr*, Δ*nprB*, Δ*vpr*, Δ*wprA*	[41]
BSG1681	(168) ΔP_srfA_	This study
BSG101	168, pBSG03 (P_srfA_-*gfp*)	This study
BSG102	168, pBSG04 (P_HpaII_-*gfp*)	This study
BSG103	BSG1681, pBSG03 (P_srfA_-*gfp*)	This study
BSG104	WB600, pBSG03 (P_srfA_-*gfp*)	This study
BSG105	WB800, pBSG03 (P_srfA_-*gfp*)	This study
BSG106	168, pBSG05 (^mut^P_srfA_-*gfp*)^a^	This study
BSG107	168, pBSG06 (^mut^P_srfA_-*ap*)	This study
Plasmids		
P7Z6	*zeo* ^r^ *bla* ^r^ *Cre/lox*	[38]
pUC19	pUC origin P_*lac*_ *Ap* ^*r*^	TaKaRa
pMA09	*E. coli*–*B. subtilis shuttle vector, Ap* ^r^ *Kan* ^r^ P_HpaII_	[30]
pMA05	*E. coli*–*B. subtilis shuttle vector, Ap* ^r^, *Kan* ^r^, P_HpaII_- *AP*	[30]
pBSG01	*bla* ^r^, *neo* ^r^, *E. coli*–*B. subtilis* shuttle vector	This work
pBSG02	pBSG01 ligated with P_srfA_	This study
pBSG03	pBSG02 with GFP ligated downstream of P_srfA_	This study
pBSG04	pBSG03 with P_srfA_ replaced by P_HpaII_	This study
pBSG05	pBSG03 with ^mut^P_srfA_	This study
pBSG06	pBSG02 with AP ligated downstream of ^mut^P_srfA_	This study

^a^ ^mut^P_srfA_: the −10 region (TAAACT) and −35 region (GTGATA) of the P_srfA_ was replaced by the consensus −10 region (TATAAT) and −35 region (TTGACT) of σ^A^-dependent promoters

### Recombinant DNA techniques

Plasmid construction was performed in *E. coli*, and extraction was processed following the standard procedure as previously described [33]. Recombinant plasmids were transformed into *B. subtilis* as previously described [30]. Enzymes were obtained from TOYOBO (Osaka, Japan), TaKaRa (Dalian, China), or New England Biolabs (Beijing, China) and were used according to the manufactures’ protocols. The primers used in this study are listed in Table 2. PCR was performed using KOD DNA polymerase (Osaka, Japan). All of the recombinant plasmids that were constructed in this work were confirmed by DNA sequencing (Shanghai Sangon Biotech Co., Ltd., China).

**Table 2:** Oligonucleotides used in this study

Primers	Sequences^a^
P1	CTCTTCCGCTTCCTCGCTCACTGACTCGC
P2	GCGGTATTTTCTCCTTACGCATCTGTGCGG
P3	GCGAGTCAGTGAGCGAGGAAGCGGAAGAGTAGAAGAAGCTTGGAGACAAGGTAAAGG
P4	CCGCACAGATGCGTAAGGAGAAAATACCGCCATATGTAAATCGCTCCTTTTTAGGTGGCAC
P5	CCCCCTTTGCTGAGGTGGCAGAGGGCAGGTATCGACAAAAATGTCATGAA
P6	CCGCACAGATGCGTAAGGAGAAAATACCGCCATTGTCATACCTCCCCTAATCTTTATAAG
P7	ACCTGCCCTCTGCCACCTCAGC
P8	GCGGTATTTTCTCCTTACGCATC
P9	CTTATAAAGATTAGGGGAGGTATGACAATGATGAGTAAAGGAGAAGAACTTTTCACTGGAG
P10	CCGCACAGATGCGTAAGGAGAAAATACCGCTTATTTGTATAGTTCATCCATG
P11	CATTGTCATACCTCCCCTAATC
P12	ATGAGTAAAGGAGAAGAACT
P13	ACTTTTCACCCATTTTTCGGTTGACAAAACATTTTTTTCATTTATAATGAACGGTAGAAAGATAAAAAATATTGAAA
P14	TTTTATCTTTCTACCGTTCATTATAAATGAAAAAAATGTTTTGTCAACCGAAAAATGGGTGAAAAGTTTCATGCGGG
P15	CTTATAAAGATTAGGGGAGGTATGACAATGATGAAAAAGCTTTTGACTGTC
P16	CCGCACAGATGCGTAAGGAGAAAATACCGCTTATTTGATATCTTCAAAAATG
P17	CGCAGATGTAGTCAACACCGAGTGCGTC
P18	ATCAAG**AGTTTACATAATATATATTCTAGGAAGT**ATCAATCAATTCCATATAGCCTTTCCC
P19	ACTGA**ACTTCCTAGAATATATATTATGTAAACT**ATGGAAATAACTTTTTACCCTTTAACGG
P20	ATCGTTGATTAGGAGATTATACGG
P21	TTGAT**ACTTCCTAGAATATATATTATGTAAACT**CTTGATATGGCTTTTTATATGTG
P22	TCCAT**AGTTTACATAATATATATTCTAGGAAGT**TCAGTCCTGCTCCTCGGCCACG

^a^Homologous sequences were underlined, and dif _*B. subtilis*_ sequence was shown in bold

### Construction of plasmids and mutant strains with markerless gene deletion

**Fig. 7:**
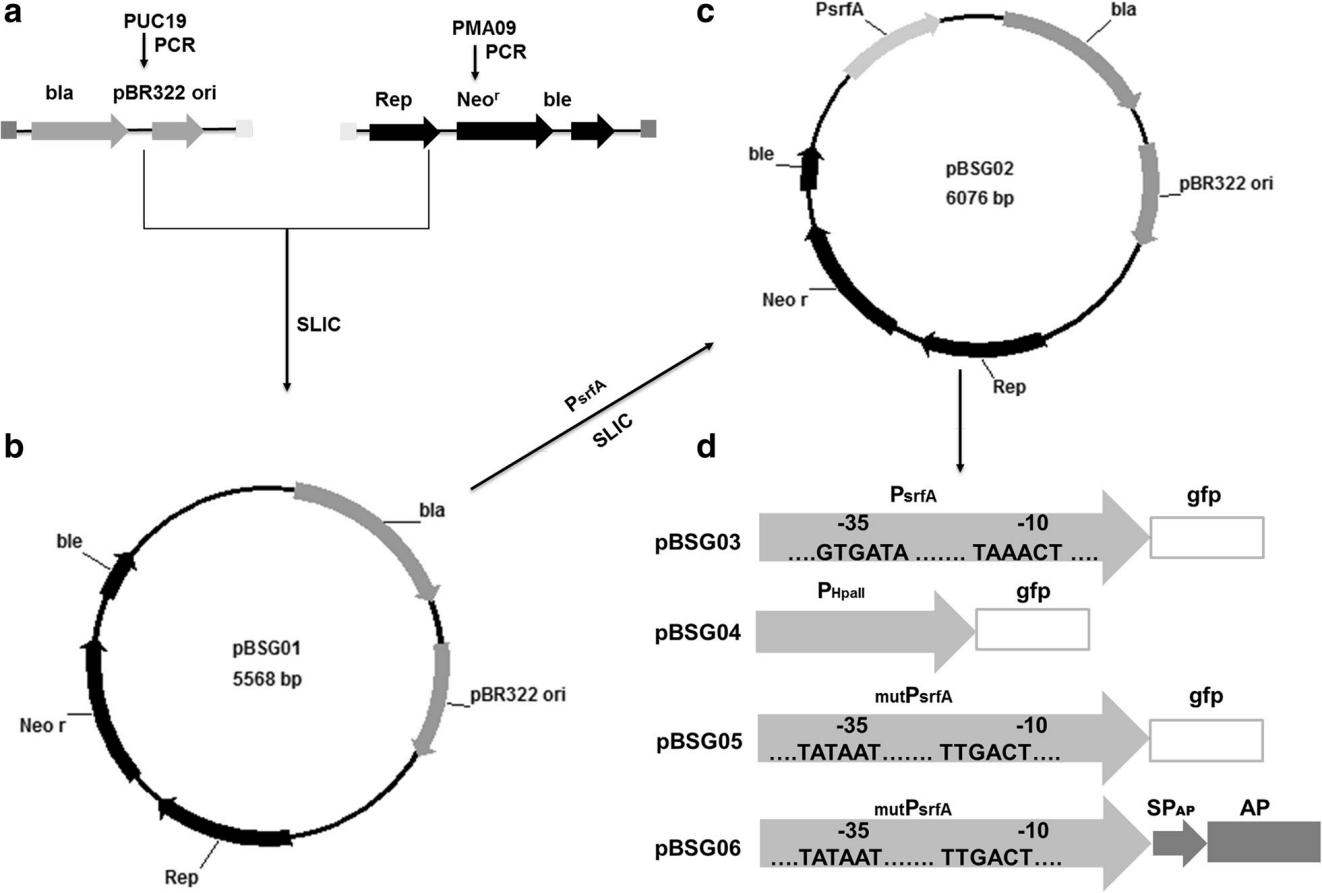
The schematic diagram of the construction of the *E. coli*–*B. subtilis* recombinant shuttle plasmids. **a** Fragment 1, harboring the ampicillin resistance gene (*bla*) and replication origin (pBR322 ori), was amplified from pUC19. Fragment 2, containing the replication protein (Rep), kanamycin resistance gene (*neo* ^r^), and bleomycin resistance gene (*ble*), was similarly obtained from pMA09 by PCR. The terminus of each of the two fragments was flanked by a 30-bp homology corresponding to each other. **b** These two fragments were fused using the Sequence and Ligation Independent Cloning (SLIC) method [35], with some modifications, and yielding Plasmid pBSG01. **c** Construction of pBSG02 with the insertion of promoter P_srfA_ into pBSG01. **d** GFP was inserted downstream of the P_srfA_, generating expression plasmid pBSG03. To compare the transcriptional strength, promoter P_HpaII_ was substituted for P_srfA_, generating plasmid pBSG04. To engineer P_sfrA_, the −10 (TAAACT) and −35 (GTGATA) motifs in P_srfA_ were replaced by the highly conserved sequence −10 (TATAAT) and −35 (TTGACT) region. The resulting plasmid was designated as pBSG05. The mutant promoter P_srfA_ was denoted as ^mut^P_srfA_

Plasmid construction was carried out using the Sequence and Ligation Independent Cloning (SLIC) method, with few modifications [34]. The primers that were used to amplify the donor sequence were designed to have 30-bp flanking regions that are homologous to the receptor plasmid insertion regions, and the receptor plasmid was linearized by inverse PCR. The main procedures used for construction are shown in Fig. 7. The plasmids constructed in this study were *E. coli*–*B. subtilis* shuttle vectors.

To construct pBSG01, Fragment 1, which is 2.2 kb and comprises the ampicillin resistance gene (*bla*) and replication region (pBR322 ori) and was flanked by the corresponding homology to Fragment 2, was amplified from pUC19 with primers P1 and P2. Concomitantly, Fragment 2 carrying the replication protein (Rep), kanamycin resistance gene (*neo*^r^), and bleomycin resistance gene (*ble*), which was flanked by the corresponding homology of Fragment 1, was amplified from plasmid pMA09 using primers P3 and P4. These two fragments were mixed at the molar ratio of 1:2 prior to treatment with T4 DNA polymerase. After a 2.5-min treatment, the mixture was incubated on ice for 10 min, allowing for the two fragments to be annealed, after which the mixture was transformed to *E. coli* JM109 competent cells [35]. The resulting plasmid was termed pBSG01.

To introduce promoter P_srfA_ into the plasmid pBSG01, the 0.6-kb P_srfA_ flanked by a 30-bp homology sequence upstream and downstream of the inserted position in pBSG01 was amplified from the chromosome of *B. subtilis* 168 using primers P5 and P6. Plasmid pBSG01 was then linearized using inverse PCR with primers P7 and P8. The two fragments were joined according to the steps described above. The resulting plasmid was termed pBSG02. To insert GFP downstream of P_srfA_ in pBSG02, the *gfp* gene was amplified from pBS1154 with primers P9 and P10, flanked by the 30-bp homology sequence corresponding to the upstream and downstream sequences at the inserted position. Meanwhile, pBSG02 was linearized by inverse PCR with primers P11 and P2. These two fragments were joined by the SLIC method mentioned above, generating plasmid pBSG03 for expression in *B. subtilis*. To construct the comparative plasmid pBSG04 under the control of promoter HpaII (P_HpaII_), P_HpaII_ was inserted into pBSG03 by replacing P_srfA_ using mega-primer PCR with a whole plasmid protocol using primers P7 and P12 [36]. Plasmid pBSG05, harboring the mutant P_srfA_, was constructed with primers P13 and P14 using pBSG03 as the template through inverse PCR. Then, the endonuclease *Dpn*I was used to cut the template plasmid. To express aminopeptidase (AP) under the control of P_srfA_, plasmid pMA05 was used as the template to amplify AP with primers P15 and P16, and pBSG02 was linearized with primers P11 and P2. Plasmid pBSG06 was obtained by processing these two fragments in the same manner as for pBSG02. All of the plasmid constructs were verified by DNA sequencing.

The markerless deletion of genes on the chromosome of *B. subtilis* 168 was performed as previously described, with some modification [37, 38]. The sequences of the *B. subtilisdif* site (dif _*B. subtilis*_: ACTTCCTAGAATATATATTATGTAAACT) were used in this study.

**Fig. 8:**
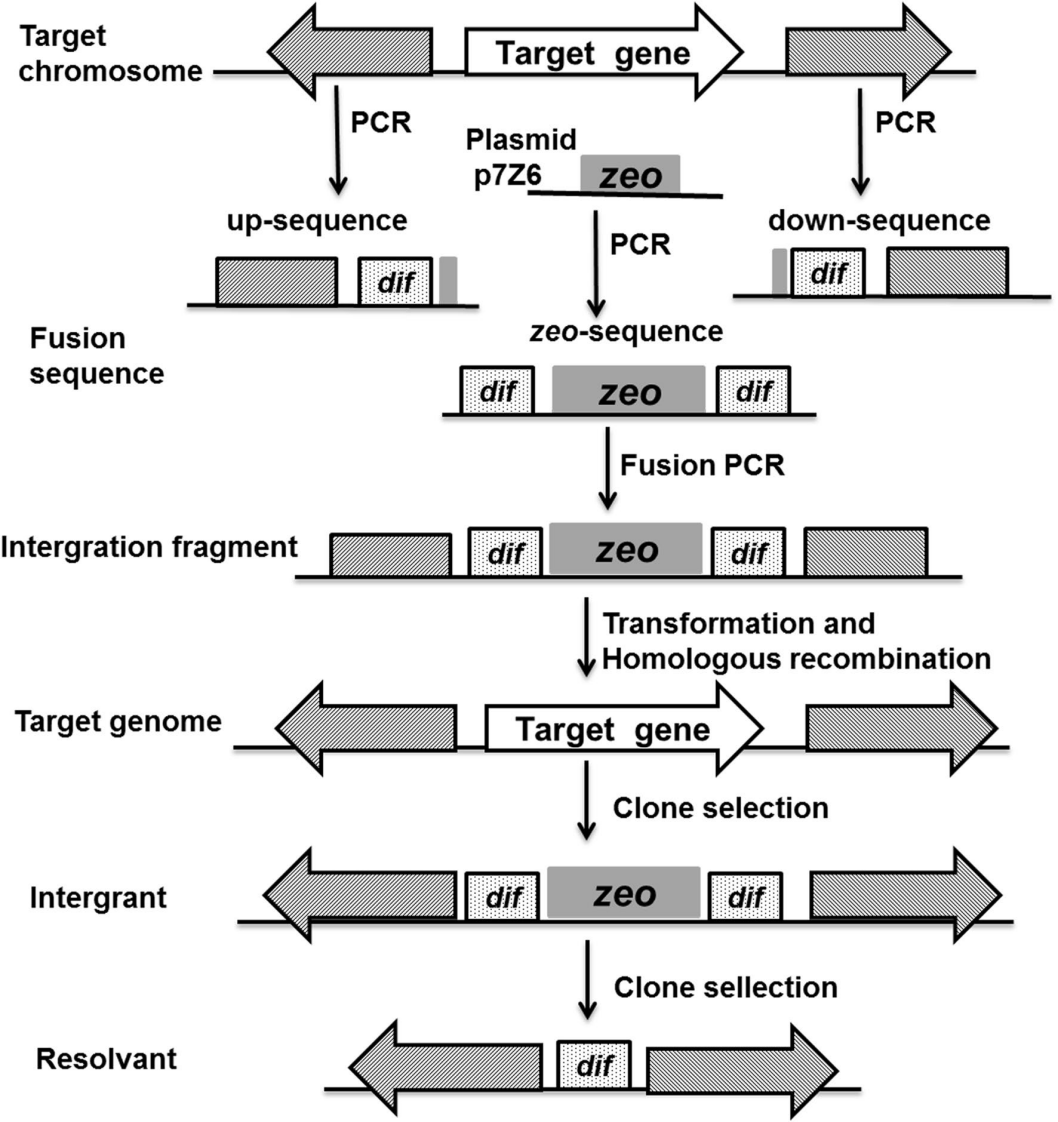
The sketch displays the procedure for the deletion of the target gene on a chromosome using the dif/Xer recombination system. The *shaded regions* represented homology between the integration cassette and the genes flanking the target gene. *zeo*, the zeocin resistance gene *zeo*. The front (up-sequence) and back (down-sequence) regions flanking the target gene to be deleted were amplified from the chromosome of *B. subtilis* 168, and the *zeo* sequence was cloned from plasmid p7Z6. These three fragments were joined by PCR; the resulting PCR products were transformed into *B. subtilis* 168, and *zeo* ^r^ transformants were selected. After subculturing, Xer recombinants were selected

To delete the promoter region in the *srf* operon from the *B. subtilis* 168 chromosome, two tripartite fragments (Fig. 8) were amplified from the *B. subtilis* genome using the primers that are listed in Table 2. The specific procedure is described as follows. The up-sequence, which consisted of the approximately 1-kb homologous fragment upstream of P_srfA_, the *dif*_*B. subtilis*_ site, and 6-bp homologous region to the 5′ end of the gene *zeo*, was amplified by PCR with primers P17 and P18. Similarly, the down-sequence, which consisted of a 5-bp homologous fragment to the 3′ end of the gene *zeo*, the *dif*_*B. subtilis*_, and the approximately 1-kb homologous fragment downstream of the gene P_srfA_, was amplified with primers P19 and P20. Next, the zeocin resistance gene *zeo* was amplified from plasmid p7Z6 using primers P21 and P22, flanked by a 5-bp homology to the sequence in the chromosome followed by the 28-bp *dif* sequence at the 5′ terminus and the 28-bp *dif* sequence followed by a 5-bp homology to the chromosome. These three fragments were assembled by fusion PCR. The resulting 2.5-kb fragment was then sequenced and transformed into the *B. subtilis* 168 competent cells. The integrants were selected on LB agar medium containing 25 μg/mL zeocin. The positive clones were selected before inoculating the LB broth in the absence of antibiotics and were incubated for approximately 36 h to produce zeocin-sensitive recombinant clones. Then, the culture was identified by replica agar plating with and without the selective antibiotic. Finally, the clones that grew on the agar plate in the absence of antibiotics were selected in liquid medium for identification by PCR using primers P17 and P20. The positive mutant strain that was deficient in P_srfA_ was named BSG1681 (*B. subtilis* 168: ΔP_srfA_).

### GFP fluorescence assay

A single colony of the appropriate *B. subtilis* strain picked on LB agar plates was inoculated in 5 mL of LB liquid medium and was cultured overnight for more than 12 h to serve as a preculture. Then, 0.6 mL of preculture was transferred into 250-mL shaking flasks that were loaded with 30 mL of LB liquid medium to cultivate for 3 days, during which the culture medium was sampled every 2 h. After cultivation, the cells were harvested by centrifugation, washed by PSB buffer (50 mM Tris–HCl, 100 mM NaCl, pH 7.5) for three times, and suspended in an appropriate diluted ratio. Then, a final volume of 200 μL of the cell suspension was transferred to each well, and the fluorescence value (expressed as Au) was recorded.

The fluorescent activity of GFP was monitored via fluorescence spectroscopy on a black flat 96-well plate using a Synergy™ H4 multimode microplate reader (BioTek Instruments, Inc., USA). The excitation wavelength was 495/9 nm, and the emission wavelength was 525/9 nm with gain 80. The determination of the GFP expression was calculated with the fluorescence intensity divided by the OD_600_ that was previously measured [39].

The relative fluorescence intensities which reflected the expression levels were calculated according to the method described by Toymentseva et al. [10]. The relative fluorescence intensity (Au/OD_600_) of three parallel samples of the recombinant *B. subtilis* strains harboring empty vector pBSG01 were averaged and subtracted from that of the recombinant strains harboring plasmids with GFP at the consistency time of cultivation. Growth was monitored by measuring the absorbance at 600 nm. The data were averaged from three independent samples of the same time points.

### Heterologous expression of aminopeptidase and enzyme activity analysis

A fresh overnight culture of the recombinant *B. subtilis* strain BSG107 harboring pBSG06 under the control of mutant P_srfA_ (^mut^P_srfA_) was inoculated into 250-mL shaking flasks that were loaded with 30 mL of LB liquid medium, the optical density of which was adjusted to OD_600_ 0.2. Then, the culture was cultivated at 37 °C with rigorous shaking for 2 days, and sampling was implemented throughout the fermentation process with an interval of 2 h. Thereafter, crude AP in the fermentation supernatant was obtained by removing recombinant *B. subtilis* cells via centrifugation (5 min, 10,000×*g*). AP activity was determined via a previously described method [23]. One unit of activity was defined as the amount of enzyme that released 1 µmol L^−1^*p*-nitroaniline min^−1^ at 37 °C (ε_405 nm_ = 9.98 L mmol^−1^ cm^−1^). The results are the averages of triplicate assays.

### Protein analysis and SDS-PAGE

After cultivation for appropriate recombinant *B. subtilis*, the cells were harvested by centrifugation. The pelleted cells were then re-suspended in PBS buffer prior to disruption by ultra-sonication on ice. The crude cell extracts were separated by centrifugation, the supernatant was analyzed by SDS-PAGE according to the standard procedure, and the gels were subsequently stained by Coomassie brilliant blue R250 staining. The concentration of the total proteins was determined by the Bradford assay and equal amounts of total protein were loaded onto SDS-PAGE gels.

## Abbreviations

P_srfA_: the promoter of the *srf* operon
PTS: phosphotransferase system
QS: quorum sensing
AP: aminopeptidase
GFP: green fluorescent protein
SLIC: sequence and ligation independent cloning
*bla*: ampicillin resistance gene
pBR322 ori: replication region
Rep: replication protein
*neo*^r^: kanamycin resistance gene
*ble*: bleomycin resistance gene
P_HpaII_: promoter of HpaII
SP_ap_: the signal peptide of aminopeptidase
^mut^P_srfA_: the mutant of promoter P_srfA_
